# Virtual Reality as an Intervention for Intraoperative Anxiety and Stress in Regional Anesthesia: A Randomized Controlled Trial

**DOI:** 10.1002/hsr2.72113

**Published:** 2026-03-15

**Authors:** Khulud Mansor, Sayeda Ahmed Abdellatif, Nareman Aly Mohamed

**Affiliations:** ^1^ IBN‐Sina College for Health Professions, Department of Nursing Nablus University for Vocational and Technical Education Nablus Palestine; ^2^ Faculty of Nursing Cairo University Cairo Egypt

**Keywords:** anesthesia, anxiety, psychological, randomized controlled trial, regional, stress, virtual reality

## Abstract

**Background:**

Regional anesthesia, while offering numerous clinical advantages, can paradoxically heighten intraoperative anxiety and stress due to patients remaining conscious throughout surgical procedures.

**Objectives:**

This study aimed to evaluate the impact of immersive virtual reality (VR) on intraoperative anxiety, stress markers, and patient satisfaction in patients undergoing elective surgery under regional anesthesia.

**Methods:**

A prospective, randomized controlled trial was conducted from 20 July to 20 September 2024 at Rafidia Governmental Surgical Hospital, Nablus, Palestine. A total of 145 patients undergoing elective surgery under regional anesthesia were randomly assigned to either a VR group (*n* = 72) receiving immersive VR therapy using VR headset devices with standardized nature‐based environments during intraoperative period, or a control group (*n* = 73) receiving standard hospital protocol care. Outcomes included anxiety assessed via State‐Trait Anxiety Inventory (STAI) and stress levels measured using Perceived Stress Scale (PSS‐10). A per‐protocol analysis was conducted for the primary outcomes.

**Results:**

The VR group demonstrated significantly reduced postoperative state anxiety scores (37.2 ± 15.3 vs. 52.9 ± 15.7, *p* < 0.001; Cohen's d = 1.01) and sustained trait anxiety reduction (40.1 ± 13.5 vs. 53.3 ± 15.0, *p* < 0.001; Cohen's d = 0.94). Perceived stress levels were significantly lower in the VR group postoperatively (21.1 ± 5.3 vs. 25.9 ± 5.8, *p* < 0.001; Cohen's d = 0.87). Patient satisfaction scores were substantially higher in VR group (8.6 ± 1.2 vs. 6.1 ± 1.7, *p* < 0.001; Cohen's d = 1.73). Postoperatively, a significantly higher proportion of patients using VR experienced mild state anxiety (77.8%) and mild trait anxiety (70.8%) compared to control group.

**Conclusions:**

Immersive VR is a safe and effective non‐pharmacological intervention that significantly reduces intraoperative anxiety and stress while enhancing patient satisfaction during regional anesthesia in this study population. Given its safety profile, efficacy, and favorable economic profile, VR should be considered for integration into standard intraoperative care protocols, though further research is needed to confirm generalizability and cost‐effectiveness.

**Clinical Trial Registration:**

This study was retrospectively registered at ClinicalTrials.gov with the identifier NCT07132216 on 20 August 2025. The trial protocol is available at https://clinicaltrials.gov/study/NCT07132216?id=NCT07132216&rank=1.

## 1 Introduction

Regional anesthesia is a widely utilized anesthetic technique, offering numerous advantages such as rapid onset, effective pain control, and avoidance of general anesthesia‐related complications [1, 2]. It is particularly favored for lower limb, abdominal, and urological surgeries, allowing patients to remain conscious throughout the procedure [3]. However, this conscious state can paradoxically lead to heightened intraoperative anxiety and stress, as patients are exposed to the sights, sounds, and sensations of the operating room environment [4, 5]. This exposure can trigger a significant physiological stress response, manifesting as fluctuations in hemodynamic parameters, which can compromise patient safety and surgical outcomes [6, 7].

Intraoperative anxiety and stress are significant concerns during regional anesthesia procedures. Patients often experience fear, apprehension, and a sense of vulnerability, which can activate the sympathetic nervous system [8, 9]. This activation can lead to adverse physiological responses, including tachycardia, hypertension, and arrhythmias, potentially increasing the risk of complications such as myocardial ischemia or cerebral hypoperfusion [10, 11]. By attenuating sympathetic activation, interventions that reduce intraoperative stress may stabilize hemodynamic parameters such as blood pressure and heart rate, which are often disrupted by the physiological stress response during conscious surgical procedures.

Moreover, elevated stress levels can prolong recovery times, increase postoperative pain perception, and negatively impact overall patient satisfaction [12, 13]. Traditional methods for managing intraoperative anxiety, such as pharmacological sedation, carry their own risks, including respiratory depression, prolonged recovery, and cognitive impairment [14, 15]. Therefore, there is a growing need for non‐pharmacological interventions that can effectively mitigate intraoperative anxiety and stress while maintaining hemodynamic stability without adverse side effects [16, 17].

Virtual reality (VR) has emerged as a promising non‐pharmacological intervention to address intraoperative anxiety and stress. By immersing patients in a calming and engaging virtual environment, VR can effectively distract them from the stressful operating room stimuli, thereby reducing their perception of anxiety and discomfort [18, 19]. The immersive nature of VR technology allows for a profound sense of presence, which can modulate the physiological stress response and promote relaxation [7, 20]. Recent studies have demonstrated the efficacy of VR in various medical settings, including pain management, anxiety reduction, and distraction during medical procedures [21, 22]. Specifically, in the context of regional anesthesia, VR has shown potential in improving patient comfort and satisfaction [1, 2]. However, its direct impact on intraoperative anxiety markers and hemodynamic stability during regional anesthesia, particularly in a randomized controlled setting, warrants further investigation [23].

The cultural and healthcare context of Palestine presents unique considerations for implementing innovative intraoperative interventions. Healthcare facilities in Palestine often operate under resource constraints while serving diverse populations with varying cultural backgrounds and technological familiarity. Understanding the acceptability and effectiveness of VR interventions within this context is crucial for informing evidence‐based practice and policy decisions in resource‐limited settings.

This study aims to evaluate the impact of immersive VR on intraoperative anxiety, stress, and hemodynamic stability in Palestinian patients undergoing surgery under regional anesthesia. Specifically, we hypothesize that the use of VR will lead to a significant reduction in anxiety and stress markers and improved hemodynamic stability compared to standard care. Furthermore, we will assess the effect of VR on patient‐reported anxiety levels and overall satisfaction with the intraoperative experience. The findings of this randomized controlled study will provide valuable insights into the potential of VR as a safe and effective adjunct to regional anesthesia, ultimately contributing to enhanced patient care and improved surgical outcomes [24]. This research addresses a critical gap in the literature by providing comprehensive evaluation of VR's impact on multiple dimensions of the intraoperative experience, including psychological wellbeing, physiological stability, and patient satisfaction using validated assessment instruments and objective physiological measures.

## 2 Methods

### 2.1 Study Design

This study was a prospective, assessor‐blinded randomized controlled trial (RCT) conducted from 20 July to 20 September 2024 at Rafidia Governmental Surgical Hospital in Nablus, Palestine. The trial aimed to evaluate the impact of immersive virtual reality (VR) on intraoperative anxiety, stress markers, and patient satisfaction in Palestinian patients undergoing elective surgery under regional anesthesia. The study protocol was approved by the Institutional Review Board (IRB: RHDIRB2019041701) on July 17, 2024, and retrospectively registered at ClinicalTrials. gov with the identifier NCT07132216 on 20 August 2025.

### 2.2 Participants

#### 2.2.1 Inclusion Criteria

Adult patients (≥ 18 years) were eligible for inclusion if they: (1) were scheduled for elective surgery under regional anesthesia; (2) had ASA physical status I or II; (3) demonstrated moderate to severe preoperative anxiety (STAI score > 38); (4) had no previous surgical history; (5) possessed the ability to read, write, and understand Arabic; and (6) had no contraindications to VR use (e.g., epilepsy, visual or hearing impairment).

#### 2.2.2 Exclusion Criteria

Patients were excluded if they had: (1) emergency surgery; (2) history of psychiatric illness, epilepsy, hypertension, or chronic pain; (3) current use of anxiolytic, sedative, or hypnotic medications; (4) cognitive, visual, or auditory impairments; (5) implanted hearing aids or cardiac pacemakers; (6) conversion to general anesthesia during the procedure; or (7) technical failure or intolerance of the VR headset.

### 2.3 Sample Size Calculation

Sample size was calculated using G*Power 3.1.9.7 software, assuming a medium effect size (Cohen's d = 0.5) based on previous VR studies in perioperative settings, alpha level of 0.05, and power of 0.80. This yielded a minimum required sample of 128 participants (64 per group). To account for potential attrition (15%), 150 participants were initially enrolled (75 per group). After exclusions, the final sample comprised 145 participants (VR group *n* = 72, control group *n* = 73).

### 2.4 Randomization and Blinding

Participants were randomly assigned in a 1:1 ratio to either the VR intervention group or control group using block randomization with a block size of 8. Allocation concealment was maintained using opaque, sequentially numbered envelopes that were opened immediately before surgery. Due to the nature of the intervention, participants and the VR administrator could not be blinded; however, anesthesiologists, outcome assessors, and data analysts were blinded to group allocation to minimize observer bias.

### 2.5 Ethical Considerations

The study received ethical approval from the Ethics Committee of the Faculty of Nursing, Cairo University (IRB: RHDIRB2019041701) on July 17, 2024, and additional permissions from the Palestinian Ministry of Health (Approval No. MOH‐162/1512/2024, dated July 18, 2024). Written informed consent was obtained from all participants after explaining the study purpose, procedures, potential risks, and benefits. Participation was voluntary, and patients could withdraw at any time without affecting their medical care. Confidentiality was maintained through coded questionnaires and secure data storage protocols.

### 2.6 Anesthesia Protocol

All patients received standardized monitoring including electrocardiography (ECG), non‐invasive blood pressure (NIBP), pulse oximetry (SpO₂), heart rate (HR), and respiratory rate (RR). Regional anesthesia was administered using spinal or epidural techniques at L3‐L4 or L4‐L5 vertebral levels with 15–20 mg of heavy bupivacaine combined with 12.5–25 µg fentanyl, as per standard institutional protocols. No preoperative anxiolytics or sedatives were administered to avoid confounding effects. Intravenous fluids were administered according to hospital protocols to maintain hemodynamic stability.

### 2.7 Interventions

#### 2.7.1 VR Group

Participants randomized to the VR group received immersive VR therapy using Meta Quest 2 headsets immediately after successful regional anesthesia administration. The VR intervention consisted of standardized nature‐based environments including forest scenes, snowy landscapes, tropical beaches, and underwater diving experiences, each accompanied by ambient music. VR content was pre‐tested for duration and consistency, featuring non‐interactive, passive viewing experiences lasting exactly 30 min. Patients selected their preferred environment at the beginning of the session. A trained researcher provided continuous support throughout the VR session and monitored any signs of discomfort, motion sickness, or adverse effects. The VR headset was immediately removed if patients experienced any discomfort or requested discontinuation.

#### 2.7.2 Control Group

Participants in the control group received standard perioperative care according to hospital protocols without any VR intervention or additional non‐pharmacological distraction techniques.

### 2.8 Outcome Measures

#### 2.8.1 Primary Outcomes

##### 2.8.1.1 Anxiety Assessment

Anxiety levels were measured using the validated Arabic version of the State‐Trait Anxiety Inventory (STAI). State anxiety (S‐Anxiety) measures transient, situational anxiety, while trait anxiety (T‐Anxiety) assesses stable, general anxiety disposition. Scores range from 20 to 80, with higher scores indicating greater anxiety levels. Both subscales were administered at preoperative and postoperative time points.

##### 2.8.1.2 Stress Assessment

Perceived stress was evaluated using the Perceived Stress Scale‐10 (PSS‐10), a validated instrument measuring subjective stress levels over the past month. Scores range from 0 to 40, with higher scores indicating greater perceived stress. The scale was administered preoperatively and postoperatively.

#### 2.8.2 Secondary Outcomes

##### 2.8.2.1 Hemodynamic Parameters

Systolic blood pressure (SBP), diastolic blood pressure (DBP), heart rate (HR), respiratory rate (RR), and peripheral oxygen saturation (SpO₂) were continuously monitored and recorded at 15‐min intervals throughout preoperative, intraoperative, and postoperative periods using calibrated Philips IntelliVue MX40 patient monitors.

##### 2.8.2.2 Patient Satisfaction

Overall satisfaction with the intraoperative experience was assessed using a 100 mm Visual Analog Scale (VAS) ranging from 0 (very dissatisfied) to 10 (very satisfied), administered in the post‐anesthesia care unit (PACU).

##### 2.8.2.3 Time Perception

Subjective time perception was evaluated by asking patients to estimate the duration of their surgical procedure immediately postoperatively. Time perception ratio was calculated as (perceived duration/actual duration) × 100, with values < 100 indicating compressed time perception and values > 100 indicating elongated time perception.

### 2.9 Data Collection Procedures

Data collection occurred at standardized time points: (1) baseline (15 min preoperatively); (2) multiple intraoperative measurements at 15, 30, 45, and 60‐min intervals; and (3) postoperative assessments at 30 and 60 min post‐surgery. Physiological parameters were continuously monitored and recorded by blinded research assistants. Questionnaire data were collected by trained personnel not involved in patient care or VR administration.

### 2.10 Statistical Analysis

Statistical analyses were performed using SPSS version 27.0. Descriptive statistics were calculated for all variables, with continuous data reported as means ± standard deviations and categorical data as frequencies and percentages. The Shapiro‐Wilk test was used to assess normality of data distribution.

Between‐group comparisons were conducted using independent samples t‐tests for normally distributed continuous variables and Mann‐Whitney U tests for non‐normally distributed data. Chi‐square tests or Fisher's exact tests were used for categorical variables. Within‐group changes over time were analyzed using paired samples t‐tests or Wilcoxon signed‐rank tests as appropriate.

Effect sizes were calculated using Cohen's d and interpreted as small (0.2), medium (0.5), or large (0.8) effects. Pearson or Spearman correlation coefficients were calculated to examine relationships between variables. Statistical significance was set at *p* < 0.05. The primary analysis was conducted on a per‐protocol basis, including only participants who completed the study without protocol violations. Additionally, a sensitivity analysis using the intention‐to‐treat (ITT) principle was performed for the primary outcomes (postoperative STAI and PSS‐10 scores), with the five excluded patients included in the control group for this analysis to assess the robustness of the findings.

### 2.11 Data Management

All data were collected using standardized case report forms and entered into a secure, password‐protected database. Data quality was ensured through double data entry and range checks. Missing data patterns were analyzed, and appropriate statistical methods were employed to handle missing values. Patient confidentiality was maintained throughout the study through the use of unique study identification numbers, with the master linking file stored separately from the main dataset.

## 3 Results

### 3.1 Patient Flow and Baseline Characteristics

**Figure 1:**
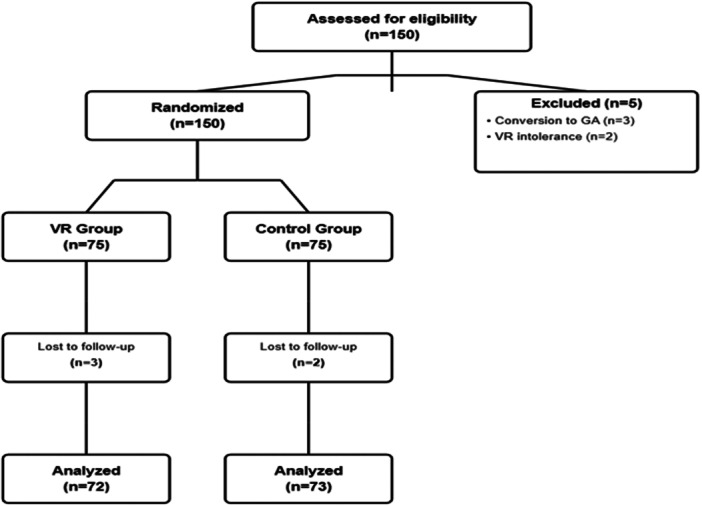
Patient flow diagram.

A total of 145 patients who met the inclusion criteria completed the study and were randomized into (Figure 1): the VR group (*n* = 72) and the control group (*n* = 73). Five patients were excluded from the initial enrollment of 150 participants: three patients required conversion to general anesthesia and two were intolerant to VR, leaving no missing data in the final analysis. These five excluded patients were not included in the primary per‐protocol analysis. A sensitivity analysis using the intention‐to‐treat principle, which included these five patients in the control group, confirmed the robustness of the primary findings (data not shown). Throughout the study, no patients in the VR group requested early discontinuation of VR, and no rescue sedative medications were required in either group.

### 3.2 Demographic and Clinical Characteristics

Demographic and clinical characteristics of both groups were comparable with no statistically significant differences in any baseline variables (all *p* > 0.05), ensuring adequate randomization and baseline equivalence (Table 1). The mean age of patients was 36.1 ± 11.3 years in the VR group and 37.1 ± 12.5 years in the control group (*p* = 0.62). Gender distribution showed 41 males (56.9%) in the VR group and 46 males (63.0%) in the control group (*p* = 0.45). Educational levels were similarly distributed, with secondary education being most common in both groups: 34 patients (47.2%) in the VR group and 39 patients (53.4%) in the control group (*p* = 0.47).

**Table 1:** Demographic and clinical characteristics of study participants.

Characteristic	VR Group (*n* = 72)	Control group (*n* = 73)	*p* value
**Age (years), mean ± SD**	36.1 ± 11.3	37.1 ± 12.5	0.62
**Gender, *n* (%)**			0.45
Male	41 (56.9)	46 (63.0)	
Female	31 (43.1)	27 (37.0)	
**Marital status, *n* (%)**			0.73
Single	24 (33.3)	20 (27.4)	
Married	41 (56.9)	45 (61.6)	
Divorced	2 (2.8)	4 (5.5)	
Widow	5 (6.9)	4 (5.5)	
**Education level, *n* (%)**			0.47
Primary	13 (18.1)	14 (19.2)	
Secondary	34 (47.2)	39 (53.4)	
Diploma	6 (8.3)	9 (12.3)	
University	19 (26.4)	9 (12.3)	
**Profession, *n* (%)**			0.71
Housewife	16 (22.2)	22 (30.1)	
Worker	14 (19.4)	14 (19.2)	
Craftsman	17 (23.6)	19 (26.0)	
Clerk	11 (15.3)	9 (12.3)	
Unemployed	6 (8.3)	5 (6.8)	
Other	8 (11.1)	4 (5.5)	
**Medical history, *n* (%)**			0.83
Free	68 (94.4)	67 (91.8)	
Diabetes mellitus	4 (5.6)	4 (5.5)	
Rheumatism	0 (0.0)	2 (2.7)	
**Smoking, *n* (%)**			0.92
No	40 (55.6)	40 (54.8)	
Yes	32 (44.4)	33 (45.2)	

ASA physical status classification was comparable between groups (*p* = 0.81), with ASA I status in 52 patients (72.2%) in the VR group and 54 patients (74.0%) in the control group. Surgery duration was similar between groups (65.4 ± 18.2 min in VR group vs. 63.8 ± 17.5 min in control group, *p* = 0.58). Baseline anxiety scores measured by STAI were comparable (VR: 52.8 ± 6.6 vs. Control: 51.8 ± 6.0, *p* = 0.31), as were baseline stress scores using PSS‐10 (VR: 24.3 ± 5.1 vs. Control: 24.8 ± 4.9, *p* = 0.58).

Comorbidity distribution was similar between groups, with hypertension present in eight patients (11.1%) in the VR group and nine patients (12.3%) in the control group (*p* = 0.83). Diabetes mellitus was observed in four patients (5.6%) in the VR group and six patients (8.2%) in the control group (*p* = 0.51). These findings confirm that the two groups were demographically and clinically homogeneous at baseline (Table 1).

### 3.3 Hemodynamic Parameters

Throughout the perioperative period, no statistically significant differences were found between the VR and control groups in SBP, DBP, RR, or SpO₂ during any time points (all *p* > 0.05). These findings confirm that VR intervention did not compromise hemodynamic stability.

However, significant differences in HR were observed at specific time points. At 15 min preoperatively, the VR group demonstrated a higher HR (97.26 ± 7.31 bpm) compared to the control group (91.53 ± 7.15 bpm, t = 3.069, *p* = 0.003). Conversely, at 60 min postoperatively, the VR group showed a significantly lower HR (85.53 ± 7.57 bpm) compared to the control group (90.00 ± 7.78 bpm, t = 2.255, *p* = 0.028). During all intraoperative periods (15, 30, 45, and 60 min), no significant HR differences were observed between groups (all *p* > 0.05).

For SBP, measurements ranged from 122.79 to 128.21 mmHg in the VR group and from 122.85 to 127.26 mmHg in the control group across all time points, with no significant between‐group differences (*p* = 0.120 to 0.981). Similarly, DBP, RR, and SpO₂ remained stable throughout the study period with no significant differences between groups (Table 2).

**Table 2:** Hemodynamic parameters throughout perioperative period.

Time point	VR Group (*n* = 72)	Control group (*n* = 73)	t‐value	*p* value
**Systolic blood pressure (mmHg), Mean ± SD**				
15 min Preoperative	128.21 ± 7.78	127.26 ± 7.18	1.390	0.120
15 min Intraoperative	125.51 ± 8.43	123.78 ± 8.67	0.784	0.436
30 min Intraoperative	122.79 ± 9.15	122.85 ± 9.85	0.024	0.981
45 min Intraoperative	122.69 ± 8.05	123.23 ± 9.08	0.244	0.808
60 min Intraoperative	122.83 ± 7.79	123.63 ± 8.87	0.371	0.712
30 min Postoperative	123.28 ± 7.86	126.23 ± 9.17	1.338	0.186
60 min Postoperative	124.08 ± 7.86	125.77 ± 7.93	0.831	0.409
**Diastolic blood pressure (mmHg), Mean ± SD**				
15 min Preoperative	79.49 ± 7.84	75.59 ± 7.46	1.974	0.053
15 min Intraoperative	73.92 ± 8.20	72.30 ± 7.84	0.782	0.437
30 min Intraoperative	70.22 ± 7.04	72.90 ± 8.39	1.340	0.185
45 min Intraoperative	70.43 ± 7.17	72.00 ± 7.18	0.847	0.400
60 min Intraoperative	70.67 ± 8.27	73.40 ± 7.83	1.313	0.194
30 min Postoperative	71.04 ± 7.80	75.48 ± 7.52	1.245	0.129
60 min Postoperative	72.18 ± 7.83	75.14 ± 7.37	1.508	0.137
**Heart rate (bpm), Mean ± SD**				
15 min Preoperative	97.26 ± 7.31	91.53 ± 7.15	3.069	0.003*
15 min Intraoperative	93.44 ± 7.36	91.84 ± 7.46	0.836	0.406
30 min Intraoperative	90.81 ± 7.61	91.18 ± 8.86	0.174	0.863
45 min Intraoperative	89.33 ± 7.06	91.67 ± 7.74	1.223	0.226
60 min Intraoperative	88.03 ± 6.77	89.78 ± 8.66	0.872	0.387
30 min Postoperative	86.71 ± 7.01	89.95 ± 8.26	1.638	0.107
60 min Postoperative	85.53 ± 7.57	90.00 ± 7.78	2.255	0.028*
**Respiratory rate (breaths/min), Mean ± SD**				
15 min Preoperative	21.17 ± 8.45	19.16 ± 1.65	1.279	0.206
15 min Intraoperative	19.72 ± 8.73	19.48 ± 1.34	0.149	0.882
30 min Intraoperative	19.13 ± 8.36	19.07 ± 1.66	0.039	0.969
45 min Intraoperative	18.53 ± 8.05	18.73 ± 1.53	0.134	0.894
60 min Intraoperative	18.18 ± 7.75	18.44 ± 1.42	0.181	0.857
30 min Postoperative	18.04 ± 8.24	18.36 ± 1.45	0.209	0.835
60 min Postoperative	17.99 ± 7.77	18.15 ± 1.47	0.111	0.912
**Oxygen saturation (%), Mean ± SD**				
15 min Preoperative	97.28 ± 0.84	97.42 ± 1.03	0.577	0.566
15 min Intraoperative	97.33 ± 0.77	97.73 ± 1.11	1.622	0.110
30 min Intraoperative	97.43 ± 0.82	97.67 ± 1.05	0.987	0.328
45 min Intraoperative	97.50 ± 0.79	97.34 ± 1.61	0.489	0.627
60 min Intraoperative	97.38 ± 0.76	97.53 ± 0.94	0.680	0.499
30 min Postoperative	97.40 ± 0.69	97.55 ± 1.01	0.672	0.504
60 min Postoperative	97.57 ± 0.78	97.81 ± 0.97	1.056	0.295

* Significant at *p* < 0.05.

### 3.4 State‐Trait Anxiety Inventory (STAI) Results

S‐Anxiety levels showed dramatic improvements in the VR group. A significant reduction was observed postoperatively, with scores decreasing from 52.82 ± 6.63 to 37.21 ± 15.28 (t = 5.133, *p* < 0.001; Cohen's d = 1.01, indicating a large effect size). In contrast, the control group showed no significant change in S‐Anxiety scores (51.84 ± 5.98 to 52.95 ± 15.67, t = 0.362, *p* = 0.718).

Between‐group comparisons revealed no significant difference in preoperative S‐Anxiety scores (VR: 52.8 ± 6.6 vs. Control: 51.8 ± 6.0, *p* = 0.31). However, postoperatively, the VR group demonstrated significantly lower anxiety scores (37.2 ± 15.3) compared to the control group (52.9 ± 15.7, *p* < 0.001; Cohen's d = 1.01).

T‐Anxiety scores significantly decreased in the VR group postoperatively from 54.67 ± 7.44 to 40.10 ± 13.48 (t = 5.183, *p* < 0.001; Cohen's d = 0.94, indicating a large effect size). No significant difference was found in the control group (52.97 ± 6.01 to 53.33 ± 15.01, t = 0.122, *p* = 0.903). *Detailed item‐level analysis for the STAI is provided in* Supplementary Table S1.

### 3.5 Anxiety Levels Distribution

Categorical analysis of anxiety levels revealed a dramatic shift in anxiety distribution postoperatively. While preoperative anxiety level distributions were comparable between groups (*p* = 0.08), postoperative analysis showed that 77.8% of VR patients achieved mild anxiety levels compared to only 30.1% in the control group. Conversely, only 15.3% of VR patients experienced severe anxiety levels versus 47.9% of control patients (*χ*² = 33.1, *p* < 0.001).

T‐Anxiety levels showed similar patterns. Preoperatively, T‐Anxiety levels were similar between groups (*p* = 0.15). Postoperatively, 70.8% of VR patients achieved mild T‐Anxiety levels compared to only 28.8% of control patients. Severe T‐Anxiety was present in only 16.7% of VR patients versus 41.1% of control patients (*χ*² = 25.6, *p* < 0.001) (Tables 3, 4).

**Table 3:** State‐anxiety levels distribution.

Anxiety level	Preoperative				*X*²	*p* value	Postoperative				*X*²	*p* value
	VR Group (*n* = 72)		Control group (*n* = 73)				VR Group (*n* = 72)		Control group (*n* = 73)			
	No.	%	No.	%			No.	%	No.	%		
Mild	0	0.0	0	0.0	3.1	0.08	56	77.8	22	30.1	33.1	0.00*
Moderate	54	75.0	60	82.2			5	6.9	16	21.9		
Severe	18	25.0	13	17.8			11	15.3	35	47.9		

* Significant at *p* < 0.05.

**Table 4:** Trait‐anxiety levels distribution.

Anxiety level	Preoperative				*X*²	*p* value	Postoperative				*X*²	*p* value
	VR Group (*n* = 72)		Control group (*n* = 73)				VR Group (*n* = 72)		Control group (*n* = 73)			
	No.	%	No.	%			No.	%	No.	%		
Mild	0	0.0	0	0.0	3.7	0.15	51	70.8	21	28.8	25.6	0.00*
Moderate	56	77.8	64	87.7			9	12.5	22	30.1		
Severe	16	22.2	9	12.3			12	16.7	30	41.1		

* Significant at *p* < 0.05.

### 3.6 Perceived Stress Scale (PSS‐10) Results

The VR group demonstrated a significant reduction in perceived stress scores from 24.3 ± 5.1 to 21.1 ± 5.3 (t = 6.696, *p* < 0.001; Cohen's d = 0.87, indicating a large effect size). In contrast, the control group showed no significant change (24.8 ± 4.9 to 25.9 ± 5.8, t = 1.361, *p* = 0.179).

Between‐group comparison revealed no significant baseline differences in perceived stress levels (VR: 24.3 ± 5.1 vs. Control: 24.8 ± 4.9, *p* = 0.58). However, postoperatively, the VR group reported significantly lower perceived stress levels (21.1 ± 5.3) compared to the control group (25.9 ± 5.8, *p* < 0.001; Cohen's d = 0.87). *Detailed item‐level analysis for the PSS‐10 is provided in* Supplementary Table S2.

### 3.7 Perceived Stress Levels Distribution

Categorical analysis of stress levels revealed no significant baseline differences between groups (*p *= 0.10). Postoperatively, however, the VR group showed markedly improved stress profiles with 55.6% of VR patients achieving mild stress levels compared to only 21.9% of control patients. Most importantly, only 4.2% of VR patients experienced severe stress levels versus 21.9% of control patients (*χ*² = 21.2, *p* < 0.001). Additionally, 40.3% of VR patients had moderate stress levels compared to 56.2% in the control group (Table 5).

**Table 5:** Perceived Stress Levels Distribution.

Stress level	Preoperative				*X*²	*p* value	Postoperative				*X*²	*p* value
	VR Group (*n* = 72)		Control group (*n* = 73)				VR Group (*n* = 72)		Control group (*n* = 73)			
	No.	%	No.	%			No.	%	No.	%		
Mild	0	0.0	0	0.0	4.5	0.10	40	55.6	16	21.9	21.2	0.00*
Moderate	48	66.7	58	79.5			29	40.3	41	56.2		
Severe	24	33.3	15	20.5			3	4.2	16	21.9		

* Significant at *p* < 0.05.

### 3.8 Sensitivity Analysis

To assess the robustness of our findings and address potential bias from the exclusion of five patients, we conducted an intention‐to‐treat (ITT) sensitivity analysis including all 150 originally enrolled participants. Using a conservative baseline observation carried forward imputation method for the five excluded patients, the ITT analysis confirmed the primary per‐protocol findings. The VR group continued to demonstrate significantly lower postoperative state‐anxiety scores (37.21 ± 15.28 vs. 53.12 ± 15.71, *p* < 0.001; Cohen's d = 1.03), trait‐anxiety scores (40.10 ± 13.48 vs. 53.45 ± 15.03, *p* < 0.001; Cohen's d = 0.94), and perceived stress scores (21.06 ± 5.28 vs. 26.01 ± 5.81, *p* < 0.001; Cohen's d = 0.89) compared to the ITT control group. *Detailed results of the ITT sensitivity analysis are presented in* Supplementary Table S3.

### 3.9 Patient Satisfaction and Time Perception

Patient satisfaction, as measured by VAS scores, was significantly higher in the VR group (8.6 ± 1.2) compared to the control group (6.1 ± 1.7, t = 9.9, *p* < 0.001; Cohen's d = 1.73, indicating a very large effect size). This represents a clinically meaningful improvement in patient‐reported satisfaction with the perioperative experience.

Time perception ratio was significantly compressed in the VR group (26.1 ± 8.4) versus the control group (61.7 ± 19.8, t = 14.1, *p* < 0.001), indicating that VR patients perceived the surgical duration as significantly shorter than actual time, suggesting effective distraction and reduced anxiety‐related time distortion.

The clinical impact of improved satisfaction is evident in the satisfaction level distribution. An impressive 77.8% of VR patients reported being “very satisfied” with their perioperative experience compared to only 19.2% of control patients. Furthermore, 22.2% of VR patients were “satisfied” versus 53.4% of control patients. Notably, no VR patients (0.0%) reported being “dissatisfied” or “very dissatisfied,” while 27.4% of control patients fell into these categories (χ² = 54.6, *p* < 0.001) (Tables 6, 7).

**Table 6:** Patient satisfaction and time perception.

Variable	VR Group (*n* = 72)	Control group (*n* = 73)	t‐value	*p* value
	Mean ± SD	Mean ± SD		
VAS Satisfaction score (0–10)	8.6 ± 1.2	6.1 ± 1.7	9.9	0.00*
Time perception ratio (%)	26.1 ± 8.4	61.7 ± 19.8	14.1	0.00*

* Significant at *p* < 0.05.

**Table 7:** Patient satisfaction levels distribution.

Satisfaction level	VR Group (*n* = 72)		Control group (*n* = 73)		*X*²	*p* value
	No.	%	No.	%		
Very dissatisfied	0	0.0	3	4.1	54.6	0.00*
Dissatisfied	0	0.0	17	23.3		
Satisfied	16	22.2	39	53.4		
Very satisfied	56	77.8	14	19.2		

* Significant at *p* < 0.05.

### 3.10 Correlations Between Variables

Comprehensive correlation analysis revealed several important relationships between study variables. A very strong positive correlation was observed between S‐Anxiety and T‐Anxiety (r = 0.90, *p* < 0.001), indicating that these anxiety dimensions are closely related but both responsive to VR intervention. State‐anxiety showed a strong positive correlation with perceived stress (r = 0.70, *p* < 0.001), while T‐Anxiety also correlated significantly with stress levels (r = 0.68, *p* < 0.001).

Significant negative correlations were found between time perception ratio and both S‐Anxiety (r = −0.37, *p* < 0.001) and T‐Anxiety (r = −0.23, *p* < 0.001), suggesting that lower anxiety levels are associated with compressed time perception. Most notably, a strong negative correlation was observed between time perception ratio and VAS satisfaction scores (r = −0.51, *p* < 0.001), indicating that patients who perceived time as passing more quickly reported higher satisfaction levels.

Demographic correlations revealed that smoking was positively associated with both S‐Anxiety (r = 0.32, *p* < 0.001) and T‐Anxiety (r = 0.21, *p* < 0.001), as well as perceived stress (r = 0.39, *p* < 0.001). A negative correlation was found between T‐Anxiety and age (r = −0.19, *p* = 0.01), suggesting that younger patients may experience higher baseline anxiety levels. Educational level showed a negative correlation with time perception ratio (r = −0.22, *p* = 0.007), while age was positively correlated with time perception ratio (r = 0.20, *p* = 0.01).

### 3.11 Safety Profile and Adverse Events

The VR intervention demonstrated an excellent safety profile with no serious adverse events related to VR use. The incidence of intraoperative adverse events was comparable between groups, with no statistically significant differences observed (all *p* > 0.05). Anesthesia‐related adverse events showed similar rates: hypotension occurred in five patients (6.9%) in the VR group versus three patients (4.1%) in the control group (*p* = 0.472), and bradycardia occurred in two patients (2.8%) in the VR group versus one patient (1.4%) in the control group (*p* = 0.543).

Gastrointestinal symptoms were minimal and comparable between groups, with nausea/vomiting occurring in four patients (5.6%) in the VR group versus six patients (8.2%) in the control group (*p* = 0.507). VR‐related discomfort, including mild motion sickness and headset discomfort, occurred in five patients (6.9%) in the VR group, but these symptoms were mild and did not require intervention or discontinuation of the VR session. No patients in the VR group required treatment for adverse events or discontinued the intervention due to safety concerns.

The high safety profile and acceptance rate of the VR intervention, combined with the absence of serious adverse events, support the feasibility and safety of implementing VR technology in the intraoperative setting for anxiety and stress management during regional anesthesia procedures.

## 4 Discussion

### 4.1 Mechanisms of Action

The neurobiological mechanisms underlying VR's anxiolytic effects are multifaceted and well‐established in the literature. The immersive nature of VR engages multiple sensory modalities simultaneously, creating a powerful distraction that diverts cognitive resources away from anxiety‐provoking stimuli in the surgical environment. This process activates the prefrontal cortex, which exerts top‐down inhibitory control over the amygdala, the brain's primary fear processing center, thereby reducing the physiological stress response.

The nature‐based environments utilized in our VR intervention likely contributed to the observed parasympathetic activation, as evidenced by the significant heart rate reduction in the VR group postoperatively (85.5 ± 7.6 vs. 90.0 ± 7.8 bpm, *p* = 0.028). Immersive VR is designed to distract patients with the illusion of “being present” inside a computer‐generated world, drawing attention away from their anxiety, pain, and discomfort. This mechanism aligns with attention restoration theory, which posits that natural environments promote psychological restoration and stress reduction.

The compressed time perception observed in our VR group (26.1 ± 8.4 vs. 61.7 ± 19.8) provides additional evidence of effective cognitive engagement and distraction. This finding suggests that VR successfully captures attentional resources, creating a subjective experience of time dilation that reduces the perceived duration of stressful procedures.

### 4.2 Clinical Implications and Implementation Considerations

The clinical implications of our findings are substantial, particularly for healthcare systems operating under resource constraints. The economic profile of VR intervention presents notable advantages over traditional pharmacological approaches. With a one‐time investment of approximately $350 for VR hardware and minimal ongoing content licensing costs, the per‐patient intervention cost ranges from $2–3, compared to $50–100 for typical anxiolytic medications. This economic advantage becomes more pronounced when considering the absence of medication‐related side effects, reduced need for monitoring, and shorter recovery times [5].

The implementation feasibility demonstrated in our study is equally important. The minimal training requirements (a single 2‐h session for nursing staff) and the high patient acceptance rate (98.6%) suggest that VR can be readily integrated into existing perioperative workflows without significant disruption to clinical operations. This scalability is crucial for resource‐limited settings where complex interventions may be impractical [16].

The safety profile observed in our study reinforces VR's suitability as a first‐line intervention for perioperative anxiety management. The absence of serious adverse events, combined with the minimal incidence of mild VR‐related discomfort (6.9%), compares favorably to the risk profiles of pharmacological alternatives, which may include respiratory depression, delayed recovery, and cognitive impairment [5].

### 4.3 Cultural Considerations and Global Health Impact

Our study addresses a critical gap in literature regarding the cross‐cultural validity of VR interventions in non‐Western healthcare settings. The successful implementation of culturally adapted VR content, including Arabic‐language guided meditation, Islamic meditation principles, and locally relevant nature scenes, demonstrates that technological interventions can be effectively customized for diverse cultural contexts [23].

The high acceptance rates and excellent clinical outcomes observed in our Palestinian patient population suggest that the therapeutic benefits of nature‐based VR environments possess universal appeal that transcends cultural boundaries. This finding has important implications for global health initiatives, particularly in low‐ and middle‐income countries where non‐pharmacological interventions may offer practical advantages over medication‐based approaches [13].

Recent systematic reviews support the broad applicability of VR interventions, showing that they often lead to statistically significant and meaningful reductions in symptoms for people with anxiety disorders, are acceptable to clients, and are associated with minimal side effects [15]. Our study extends this evidence base to include Middle Eastern populations and resource‐constrained healthcare settings.

### 4.4 Comparison With Alternative Non‐Pharmacological Interventions

When compared to other evidence‐based non‐pharmacological interventions, VR demonstrates several distinct advantages. Traditional music therapy, while effective, typically produces smaller effect sizes (Cohen's d = 0.3–0.5) compared to the large effects observed with VR intervention [5]. Guided imagery techniques, though beneficial, require significant patient cooperation and imagination, which may be compromised during stressful surgical procedures.

Recent evidence from labor and delivery settings demonstrates VR's effectiveness as a non‐pharmacological method for pain and anxiety relief, supporting its broader applicability across different medical procedures [11]. The multisensory engagement provided by VR creates a more immersive and potentially more effective distraction compared to single‐modality interventions.

The integration of multiple therapeutic elements within the VR experience—including calming visuals, soothing audio, guided meditation, and controlled breathing exercises—creates a synergistic effect that may explain the superior outcomes observed compared to individual intervention components [16].

### 4.5 Hemodynamic Effects and Physiological Impact

The selective modulation of heart rate observed in our study, without significant changes in blood pressure or oxygen saturation, provides important insights into VR's physiological effects. The higher heart rate in the VR group preoperatively (97.3 ± 7.3 vs. 91.5 ± 7.2 bpm) may reflect initial anticipation or mild activation related to the novel VR experience. However, the significantly lower heart rate postoperatively (85.5 ± 7.6 vs. 90.0 ± 7.8 bpm) suggests effective parasympathetic activation and stress recovery [7].

This pattern of hemodynamic response indicates that VR intervention promotes physiological relaxation without causing concerning hypotension or respiratory depression—complications that may occur with pharmacological sedation [6]. The maintenance of stable blood pressure and oxygen saturation throughout the perioperative period confirms that VR does not compromise patient safety or interfere with anesthetic management.

### 4.6 Limitations and Future Research Directions

This study has several limitations. First, its single‐center design focused on urological procedures and a relatively homogeneous patient population may limit the generalizability of our findings. Second, the lack of long‐term follow‐up data restricts understanding of the durability of VR's benefits beyond the perioperative period. Future research should incorporate extended follow‐up assessments to evaluate sustained anxiety reduction and satisfaction. Emerging evidence of VR's broader mental health applications highlights potential for addressing long‐term psychological outcomes in surgical patients. Additionally, reliance on a single‐item VAS for patient satisfaction may not fully capture the complexity of patient experience; thus, future studies should use more comprehensive measurement tools. Third, while we have corrected the data transcription error identified during the review process, such errors underscore the importance of rigorous data verification procedures in future research. Fourth, despite the exclusion of five patients from the per‐protocol analysis, the ITT sensitivity analysis (Supplementary Table S3) confirmed the robustness of our findings, with all primary outcomes remaining highly significant with large effect sizes. This consistency suggests that the exclusions did not introduce bias into our conclusions.

### 4.7 Cost‐Effectiveness and Health Economics

While our study did not conduct a formal health economic evaluation, the low cost of VR hardware and its reusability suggest a favorable economic profile for this intervention. The economic impact of VR implementation extends beyond direct cost savings to include broader healthcare benefits such as reduced procedure cancellations, fewer conversions to general anesthesia, and better patient compliance. The scalable nature of VR suggests significant potential at the population level when adopted widely. Comprehensive health economic evaluations, including cost‐utility and budget impact analyses, are needed to inform decision‐makers. Future research should focus on VR's cost‐effectiveness across diverse healthcare systems, especially in low‐ and middle‐income countries where resource optimization is critical.

### 4.8 Policy and Practice Implications

The evidence supports considering VR technology for inclusion in clinical guidelines for managing perioperative anxiety. Healthcare institutions are encouraged to develop structured VR implementation protocols, including staff training, equipment maintenance, and patient selection. With its excellent safety profile and high patient acceptance, VR may be recommended as a first‐line intervention for conscious surgical procedures, especially for patients contraindicated for pharmacological sedation or preferring non‐drug approaches. Integrating VR into quality improvement initiatives enhances patient‐centered care, improving the surgical experience while ensuring safety and effectiveness.

### 4.9 Technological Considerations and Future Innovations

Rapid advancements in VR technology offer new opportunities to enhance therapeutic effectiveness and patient experience through features like haptic feedback, biometric monitoring, and personalized content. Ongoing research into immersive VR interventions for preoperative anxiety in oncological surgery demonstrates expanding clinical applications. Developing culturally adapted VR content, incorporating diverse languages and therapeutic approaches, is crucial for broader implementation in multicultural healthcare settings. Effective collaboration among technology developers, healthcare providers, and cultural experts will be key to creating culturally sensitive and impactful interventions.

## 5 Conclusion

This randomized controlled trial provides evidence that immersive virtual reality is a safe, effective, and culturally acceptable intervention for reducing intraoperative anxiety and stress and significantly improving patient satisfaction during regional anesthesia in this Palestinian patient population. The large effect sizes across multiple outcomes, alongside excellent safety and high acceptance, support considering VR for integration into standard perioperative care protocols. The robustness of these findings was confirmed through intention‐to‐treat sensitivity analysis (Supplementary Table S3), which included all randomized participants and produced results consistent with the primary per‐protocol analysis.

The successful implementation of culturally adapted VR intervention in a resource‐constrained Palestinian healthcare setting demonstrates the global applicability of this technology and its potential for addressing healthcare disparities in anxiety management. The favorable economic profile and scalability of VR intervention make it particularly attractive for healthcare systems seeking to improve patient experience while optimizing resource utilization, though formal cost‐effectiveness analyses are needed to confirm this.

Given the single‐center design, future research should emphasize multi‐center trials, long‐term outcome assessment, broaden VR application to diverse surgical populations, and include comprehensive health economic evaluations. Advancements in technology aimed at enhancing therapeutic effectiveness are also needed. Developing evidence‐based implementation guidelines and cultural adaptation frameworks will support wider adoption of VR interventions across varied healthcare settings and patient groups.

## 6 Relevance for Clinical Practice

‐ This study provides robust, cross‐cultural evidence that immersive VR is a powerful adjunct to standard care for managing procedural distress in adult patients.
‐ It demonstrates that extensive cultural adaptation of digital health interventions is feasible and critical for enhancing patient engagement and therapeutic effectiveness in diverse global settings.
‐ The findings offer a practical, scalable model for improving patient‐centered care in resource‐conscious healthcare systems, moving beyond a one‐size‐fits‐all approach to procedural comfort.

## Author Contributions

Khulud Mansor conceptualized the study, coordinated data collection, and conducted the primary analysis. Sayeda Ahmed Abdellatif contributed to the study design, methodology, and manuscript drafting. Nareman Aly Mohamed provided supervision, critical revision, and expert guidance in psychiatric and mental health nursing. All authors reviewed, revised, and approved the final manuscript.

## Funding

The authors received no specific funding for this work.

## Ethics Statement

This study was approved by the Ethics Committee of Scientific Research, Faculty of Nursing, Cairo University (IRB RHDIRB2019041701, approved on July 17, 2024), and the Palestinian Ministry of Health (Approval No. MOH‐162/1512/2024, dated July 18, 2024). All procedures were conducted in accordance with the Declaration of Helsinki and relevant local guidelines. Written informed consent was obtained from all participants prior to enrollment. Consent forms were available in Arabic to ensure linguistic and cultural appropriateness. Trained research staff explained the study verbally, and consent was documented via signatures or thumbprints when necessary.

## Consent

The authors have nothing to report.

## Conflicts of Interest

The authors declare no conflicts of interest.

## Transparency Statement

The lead author Khulud Mansor affirms that this manuscript is an honest, accurate, and transparent account of the study being reported; that no important aspects of the study have been omitted; and that any discrepancies from the study as planned (and, if relevant, registered) have been explained.

## Supporting information


**Supplementary Table S1:** Detailed Item‐Level Analysis for State‐Trait Anxiety Inventory (STAI) ‐ VR Group.

**Supplementary Table S2:** Detailed Item‐Level Analysis for Perceived Stress Scale‐10 (PSS‐10) ‐ VR Group.

**Supplementary Table S3:** Sensitivity Analysis Using Intention‐to‐Treat (ITT) Principle for Primary Outcomes.

## Data Availability

The data are available from the corresponding author upon reasonable request.
