# Supplementary material for: Virtual Reality as an Intervention for Intraoperative Anxiety and Stress in Regional Anesthesia: A Randomized Controlled Trial
Source: Health Sci Rep. 2026 Mar 15;9(3):e72113. doi: 10.1002/hsr2.72113 (PMC13097521; doi:10.1002/hsr2.72113)
Supplement: Supplementary file 1 — Supplementary Table S1: Detailed Item‐Level Analysis for State‐Trait Anxiety Inventory (STAI) ‐ VR Group. [file HSR2-9-e72113-s001.docx]

**Supplementary Table S1: Detailed Item-Level Analysis for State-Trait Anxiety Inventory (STAI) - VR Group**

| **Item** | **Preoperative Mean ± SD** | **Postoperative Mean ± SD** | **t-value** | **p-value** |
| --- | --- | --- | --- | --- |
| **State-Anxiety Items** |  |  |  |  |
| 1. I feel calm | 3.15 ± 0.64 | 1.90 ± 1.02 | 5.686 | <0.001* |
| 2. I feel secure | 2.99 ± 0.59 | 1.90 ± 0.87 | 5.679 | <0.001* |
| 3. I feel tense | 3.53 ± 0.56 | 2.17 ± 1.06 | 6.214 | <0.001* |
| 4. I feel strained | 2.81 ± 0.80 | 1.93 ± 0.84 | 4.155 | <0.001* |
| 5. I feel at ease | 3.10 ± 0.59 | 1.92 ± 0.85 | 6.246 | <0.001* |
| 6. I feel upset | 3.07 ± 0.64 | 1.88 ± 0.99 | 5.529 | <0.001* |
| 7. I am presently worrying | 3.14 ± 0.76 | 2.03 ± 0.93 | 5.062 | <0.001* |
| 8. I feel satisfied | 3.22 ± 0.65 | 1.97 ± 0.92 | 6.078 | <0.001* |
| 9. I feel frightened | 3.40 ± 0.57 | 2.07 ± 0.97 | 6.475 | <0.001* |
| 10. I feel comfortable | 2.94 ± 0.67 | 1.74 ± 0.87 | 5.986 | <0.001* |
| 11. I feel self-confident | 2.22 ± 0.98 | 1.65 ± 0.73 | 2.555 | 0.013* |
| 12. I feel nervous | 3.25 ± 0.78 | 1.86 ± 1.01 | 5.966 | <0.001* |
| 13. I feel jittery | 2.32 ± 0.92 | 1.44 ± 0.90 | 3.745 | <0.001* |
| 14. I feel indecisive | 2.74 ± 0.93 | 1.57 ± 1.03 | 4.618 | <0.001* |
| 15. I am relaxed | 3.14 ± 0.51 | 1.96 ± 1.01 | 5.712 | <0.001* |
| 16. I feel content | 2.26 ± 0.67 | 1.61 ± 0.78 | 3.462 | 0.001* |
| 17. I am worried | 3.07 ± 0.51 | 1.93 ± 1.08 | 5.228 | <0.001* |
| 18. I feel confused | 2.26 ± 0.77 | 1.44 ± 0.80 | 4.045 | <0.001* |
| 19. I feel steady | 2.90 ± 0.63 | 2.19 ± 0.80 | 3.819 | <0.001* |
| 20. I feel pleasant | 2.31 ± 0.90 | 2.04 ± 0.85 | 1.195 | 0.237 |
| **State-Anxiety Total Score** | **52.82 ± 6.63** | **37.21 ± 15.28** | **5.133** | **<0.001*** |
| **Trait-Anxiety Items** |  |  |  |  |
| 1. I feel pleasant | 2.89 ± 0.71 | 2.01 ± 0.92 | 4.221 | <0.001* |
| 2. I tire quickly | 2.76 ± 0.68 | 1.98 ± 0.88 | 3.987 | <0.001* |
| 3. I feel like crying | 2.91 ± 0.73 | 2.11 ± 0.95 | 4.112 | <0.001* |
| 4. I wish I could be as happy as others | 2.84 ± 0.69 | 2.08 ± 0.91 | 3.876 | <0.001* |
| 5. I am losing out on things | 2.67 ± 0.72 | 1.89 ± 0.87 | 4.023 | <0.001* |
| 6. I feel rested | 2.73 ± 0.65 | 2.03 ± 0.84 | 3.654 | <0.001* |
| 7. I am calm, cool, and collected | 2.81 ± 0.68 | 2.12 ± 0.89 | 3.789 | <0.001* |
| 8. I feel difficulties are piling up | 2.94 ± 0.77 | 2.21 ± 0.96 | 4.234 | <0.001* |
| 9. I worry too much over things | 2.88 ± 0.71 | 2.15 ± 0.93 | 4.001 | <0.001* |
| 10. I am happy | 2.62 ± 0.69 | 1.92 ± 0.85 | 3.567 | <0.001* |
| 11. I have disturbing thoughts | 2.79 ± 0.73 | 2.05 ± 0.91 | 3.912 | <0.001* |
| 12. I lack self-confidence | 2.68 ± 0.70 | 1.94 ± 0.88 | 3.778 | <0.001* |
| 13. I feel secure | 2.71 ± 0.66 | 2.01 ± 0.86 | 3.654 | <0.001* |
| 14. I make decisions easily | 2.58 ± 0.68 | 1.89 ± 0.84 | 3.445 | 0.001* |
| 15. I feel inadequate | 2.73 ± 0.71 | 2.04 ± 0.89 | 3.567 | <0.001* |
| 16. I am content | 2.61 ± 0.67 | 1.93 ± 0.86 | 3.389 | 0.001* |
| 17. Some unimportant thoughts run through my mind | 2.77 ± 0.72 | 2.08 ± 0.92 | 3.678 | <0.001* |
| 18. I take disappointments so keenly | 2.69 ± 0.69 | 2.01 ± 0.88 | 3.456 | 0.001* |
| 19. I am steady | 2.63 ± 0.66 | 1.95 ± 0.85 | 3.345 | 0.001* |
| 20. I get tense as I think over recent concerns | 2.86 ± 0.74 | 2.16 ± 0.94 | 4.112 | <0.001* |
| **Trait-Anxiety Total Score** | **54.67 ± 7.44** | **40.10 ± 13.48** | **5.183** | **<0.001*** |

*Significant at p < 0.05

**Supplementary Table S1 (continued): Detailed Item-Level Analysis for State-Trait Anxiety Inventory (STAI) - Control Group**

| **Item** | **Preoperative Mean ± SD** | **Postoperative Mean ± SD** | **t-value** | **p-value** |
| --- | --- | --- | --- | --- |
| **State-Anxiety Items** |  |  |  |  |
| 1. I feel calm | 3.11 ± 0.61 | 3.15 ± 0.68 | 0.234 | 0.815 |
| 2. I feel secure | 2.95 ± 0.57 | 3.01 ± 0.63 | 0.345 | 0.731 |
| 3. I feel tense | 3.48 ± 0.54 | 3.52 ± 0.61 | 0.289 | 0.773 |
| 4. I feel strained | 2.78 ± 0.77 | 2.84 ± 0.82 | 0.312 | 0.756 |
| 5. I feel at ease | 3.06 ± 0.56 | 3.11 ± 0.62 | 0.278 | 0.782 |
| 6. I feel upset | 3.03 ± 0.62 | 3.08 ± 0.68 | 0.301 | 0.765 |
| 7. I am presently worrying | 3.11 ± 0.73 | 3.16 ± 0.79 | 0.289 | 0.773 |
| 8. I feel satisfied | 3.18 ± 0.63 | 3.23 ± 0.69 | 0.312 | 0.756 |
| 9. I feel frightened | 3.36 ± 0.55 | 3.41 ± 0.61 | 0.267 | 0.790 |
| 10. I feel comfortable | 2.91 ± 0.65 | 2.96 ± 0.71 | 0.301 | 0.765 |
| 11. I feel self-confident | 2.19 ± 0.95 | 2.24 ± 1.01 | 0.234 | 0.815 |
| 12. I feel nervous | 3.22 ± 0.76 | 3.27 ± 0.82 | 0.278 | 0.782 |
| 13. I feel jittery | 2.29 ± 0.90 | 2.34 ± 0.96 | 0.245 | 0.807 |
| 14. I feel indecisive | 2.71 ± 0.91 | 2.76 ± 0.97 | 0.234 | 0.815 |
| 15. I am relaxed | 3.11 ± 0.49 | 3.16 ± 0.55 | 0.312 | 0.756 |
| 16. I feel content | 2.23 ± 0.65 | 2.28 ± 0.71 | 0.289 | 0.773 |
| 17. I am worried | 3.04 ± 0.49 | 3.09 ± 0.55 | 0.301 | 0.765 |
| 18. I feel confused | 2.23 ± 0.75 | 2.28 ± 0.81 | 0.267 | 0.790 |
| 19. I feel steady | 2.87 ± 0.61 | 2.92 ± 0.67 | 0.289 | 0.773 |
| 20. I feel pleasant | 2.28 ± 0.88 | 2.33 ± 0.94 | 0.234 | 0.815 |
| **State-Anxiety Total Score** | **51.84 ± 5.98** | **52.95 ± 15.67** | **0.362** | **0.718** |
| **Trait-Anxiety Total Score** | **52.97 ± 6.01** | **53.33 ± 15.01** | **0.122** | **0.903** |
