# Supplementary material for: Virtual Reality as an Intervention for Intraoperative Anxiety and Stress in Regional Anesthesia: A Randomized Controlled Trial
Source: Health Sci Rep. 2026 Mar 15;9(3):e72113. doi: 10.1002/hsr2.72113 (PMC13097521; doi:10.1002/hsr2.72113)
Supplement: Supplementary file 2 — Supplementary Table S2: Detailed Item‐Level Analysis for Perceived Stress Scale‐10 (PSS‐10) ‐ VR Group. [file HSR2-9-e72113-s002.docx]

**Supplementary Table S2: Detailed Item-Level Analysis for Perceived Stress Scale-10 (PSS-10) - VR Group**

| **Item** | **Preoperative Mean ± SD** | **Postoperative Mean ± SD** | **t-value** | **p-value** |
| --- | --- | --- | --- | --- |
| 1. In the last month, how often have you been upset because of something that happened unexpectedly? | 2.41 ± 0.65 | 2.01 ± 0.91 | 2.988 | 0.004* |
| 2. In the last month, how often have you felt that you were unable to control the important things in your life? | 2.60 ± 0.66 | 1.94 ± 0.85 | 3.359 | 0.001* |
| 3. In the last month, how often have you felt nervous and "stressed"? | 2.62 ± 0.60 | 2.01 ± 0.92 | 4.039 | <0.001* |
| 4. In the last month, how often have you felt confident about your ability to handle your personal problems? | 2.90 ± 1.19 | 2.10 ± 1.05 | 2.761 | 0.008* |
| 5. In the last month, how often have you felt that things were going your way? | 2.54 ± 1.76 | 2.51 ± 1.20 | 0.077 | 0.939 |
| 6. In the last month, how often have you found that you could not cope with all the things that you had to do? | 2.54 ± 0.53 | 1.67 ± 0.84 | 4.798 | <0.001* |
| 7. In the last month, how often have you been able to control irritations in your life? | 3.28 ± 1.10 | 2.18 ± 1.07 | 3.926 | <0.001* |
| 8. In the last month, how often have you felt that you were on top of things? | 2.79 ± 1.28 | 2.39 ± 1.08 | 1.308 | 0.196 |
| 9. In the last month, how often have you been angered because of things that were outside of your control? | 2.81 ± 0.76 | 1.68 ± 1.06 | 4.745 | <0.001* |
| 10. In the last month, how often have you felt difficulties were piling up so high that you could not overcome them? | 2.79 ± 0.69 | 1.68 ± 0.99 | 5.038 | <0.001* |
| **PSS-10 Total Score** | **24.3 ± 5.1** | **21.1 ± 5.3** | **6.696** | **<0.001*** |

**Significant at p < 0.05
*Note: Items 4, 5, 7, and 8 are reverse-scored. Scores presented are after reverse scoring.*

**Supplementary Table S2 (continued): Detailed Item-Level Analysis for Perceived Stress Scale-10 (PSS-10) - Control Group**

| **Item** | **Preoperative Mean ± SD** | **Postoperative Mean ± SD** | **t-value** | **p-value** |
| --- | --- | --- | --- | --- |
| 1. In the last month, how often have you been upset because of something that happened unexpectedly? | 2.45 ± 0.63 | 2.51 ± 0.68 | 0.345 | 0.731 |
| 2. In the last month, how often have you felt that you were unable to control the important things in your life? | 2.64 ± 0.64 | 2.69 ± 0.69 | 0.301 | 0.765 |
| 3. In the last month, how often have you felt nervous and "stressed"? | 2.66 ± 0.58 | 2.71 ± 0.63 | 0.312 | 0.756 |
| 4. In the last month, how often have you felt confident about your ability to handle your personal problems? | 2.94 ± 1.15 | 2.99 ± 1.20 | 0.234 | 0.815 |
| 5. In the last month, how often have you felt that things were going your way? | 2.58 ± 1.72 | 2.63 ± 1.77 | 0.189 | 0.851 |
| 6. In the last month, how often have you found that you could not cope with all the things that you had to do? | 2.58 ± 0.51 | 2.63 ± 0.56 | 0.312 | 0.756 |
| 7. In the last month, how often have you been able to control irritations in your life? | 3.32 ± 1.07 | 3.37 ± 1.12 | 0.267 | 0.790 |
| 8. In the last month, how often have you felt that you were on top of things? | 2.83 ± 1.24 | 2.88 ± 1.29 | 0.234 | 0.815 |
| 9. In the last month, how often have you been angered because of things that were outside of your control? | 2.85 ± 0.74 | 2.90 ± 0.79 | 0.289 | 0.773 |
| 10. In the last month, how often have you felt difficulties were piling up so high that you could not overcome them? | 2.83 ± 0.67 | 2.88 ± 0.72 | 0.301 | 0.765 |
| **PSS-10 Total Score** | **24.8 ± 4.9** | **25.9 ± 5.8** | **1.361** | **0.179** |

**Note: Items 4, 5, 7, and 8 are reverse-scored. Scores presented are after reverse scoring.*

**Supplementary Table S3: Sensitivity Analysis Using Intention-to-Treat (ITT) Principle for Primary Outcomes**

| **Outcome** | **VR Group (n=72)** | **Control Group (n=78)*** | **Mean Difference (95% CI)** | **t-value** | **p-value** |
| --- | --- | --- | --- | --- | --- |
| Postoperative State-Anxiety Score | 37.21 ± 15.28 | 53.12 ± 15.71 | -15.91 (-19.82 to -12.00) | 8.234 | <0.001* |
| Postoperative Trait-Anxiety Score | 40.10 ± 13.48 | 53.45 ± 15.03 | -13.35 (-17.12 to -9.58) | 7.891 | <0.001* |
| Postoperative PSS-10 Score | 21.06 ± 5.28 | 26.01 ± 5.81 | -4.95 (-6.72 to -3.18) | 5.567 | <0.001* |

**Significant at p < 0.05
**Control group for ITT analysis includes the original 73 control participants plus 5 patients excluded from the per-protocol analysis (3 converted to general anesthesia, 2 intolerant to VR), with their outcomes imputed using baseline observation carried forward method.**
