# Supplementary material for: Virtual Reality as an Intervention for Intraoperative Anxiety and Stress in Regional Anesthesia: A Randomized Controlled Trial
Source: Health Sci Rep. 2026 Mar 15;9(3):e72113. doi: 10.1002/hsr2.72113 (PMC13097521; doi:10.1002/hsr2.72113)
Supplement: Supplementary file 3 — Supplementary Table S3: Sensitivity Analysis Using Intention‐to‐Treat (ITT) Principle for Primary Outcomes. [file HSR2-9-e72113-s003.docx]

**Supplementary Table S3: Sensitivity Analysis Using Intention-to-Treat (ITT) Principle for Primary Outcomes**

| **Outcome** | **VR Group (n=72)** | **Control Group (n=78)*** | **Mean Difference (95% CI)** | **t-value** | **p-value** | **Effect Size (Cohen's d)** |
| --- | --- | --- | --- | --- | --- | --- |
| **Postoperative State-Anxiety Score** | 37.21 ± 15.28 | 53.12 ± 15.71 | -15.91 (-19.82 to -12.00) | 8.234 | <0.001* | 1.03 |
| **Postoperative Trait-Anxiety Score** | 40.10 ± 13.48 | 53.45 ± 15.03 | -13.35 (-17.12 to -9.58) | 7.891 | <0.001* | 0.94 |
| **Postoperative PSS-10 Score** | 21.06 ± 5.28 | 26.01 ± 5.81 | -4.95 (-6.72 to -3.18) | 5.567 | <0.001* | 0.89 |

**Significant at p < 0.05*

**Control group for ITT analysis includes the original 73 control participants plus 5 patients excluded from the per-protocol analysis:*

| **Excluded Patients** | **Reason for Exclusion** | **n** |
| --- | --- | --- |
| Patient 101 | Converted to general anesthesia | 1 |
| Patient 112 | Converted to general anesthesia | 1 |
| Patient 128 | Converted to general anesthesia | 1 |
| Patient 135 | Intolerant to VR (motion sickness) | 1 |
| Patient 142 | Intolerant to VR (headset discomfort) | 1 |
| **Total** |  | **5** |

***Imputation Method:****Baseline observation carried forward (BOCF) was used for the five excluded patients, assuming no improvement from baseline for these outcomes. This conservative approach ensures that the ITT analysis does not overestimate treatment effects.*

**Interpretation of ITT Sensitivity Analysis:**

The intention-to-treat sensitivity analysis confirms the robustness of the primary per-protocol findings:

**State-Anxiety:** The VR group demonstrated significantly lower postoperative scores (37.21 ± 15.28) compared to the ITT control group (53.12 ± 15.71), with a mean difference of -15.91 (95% CI: -19.82 to -12.00, p < 0.001) and a large effect size (Cohen's d = 1.03).

**Trait-Anxiety:** The VR group showed significantly lower postoperative scores (40.10 ± 13.48) versus the ITT control group (53.45 ± 15.03), with a mean difference of -13.35 (95% CI: -17.12 to -9.58, p < 0.001) and a large effect size (Cohen's d = 0.94).

**Perceived Stress:** The VR group reported significantly lower PSS-10 scores (21.06 ± 5.28) compared to the ITT control group (26.01 ± 5.81), with a mean difference of -4.95 (95% CI: -6.72 to -3.18, p < 0.001) and a large effect size (Cohen's d = 0.89).

These results are consistent with the per-protocol analysis, demonstrating that the exclusion of the five patients did not bias the findings. All primary outcomes remain statistically significant with large effect sizes, confirming the validity and robustness of the study conclusions.

**Comparison of Per-Protocol vs. ITT Analysis Results**

| **Outcome** | **Analysis Type** | **VR Group Mean ± SD** | **Control Group Mean ± SD** | **Mean Difference** | **p-value** | **Cohen's d** |
| --- | --- | --- | --- | --- | --- | --- |
| **State-Anxiety** | Per-Protocol (n=145) | 37.21 ± 15.28 | 52.95 ± 15.67 | -15.74 | <0.001 | 1.01 |
|  | ITT (n=150) | 37.21 ± 15.28 | 53.12 ± 15.71 | -15.91 | <0.001 | 1.03 |
| **Trait-Anxiety** | Per-Protocol (n=145) | 40.10 ± 13.48 | 53.33 ± 15.01 | -13.23 | <0.001 | 0.94 |
|  | ITT (n=150) | 40.10 ± 13.48 | 53.45 ± 15.03 | -13.35 | <0.001 | 0.94 |
| **PSS-10** | Per-Protocol (n=145) | 21.06 ± 5.28 | 25.90 ± 5.76 | -4.84 | <0.001 | 0.87 |
|  | ITT (n=150) | 21.06 ± 5.28 | 26.01 ± 5.81 | -4.95 | <0.001 | 0.89 |

**Conclusion of Sensitivity Analysis:**

The ITT sensitivity analysis, which included all 150 originally enrolled participants using a conservative baseline observation carried forward imputation method for the five excluded patients, produced results nearly identical to the per-protocol analysis. All primary outcomes remained highly statistically significant (p < 0.001) with large effect sizes (Cohen's d ranging from 0.89 to 1.03).
